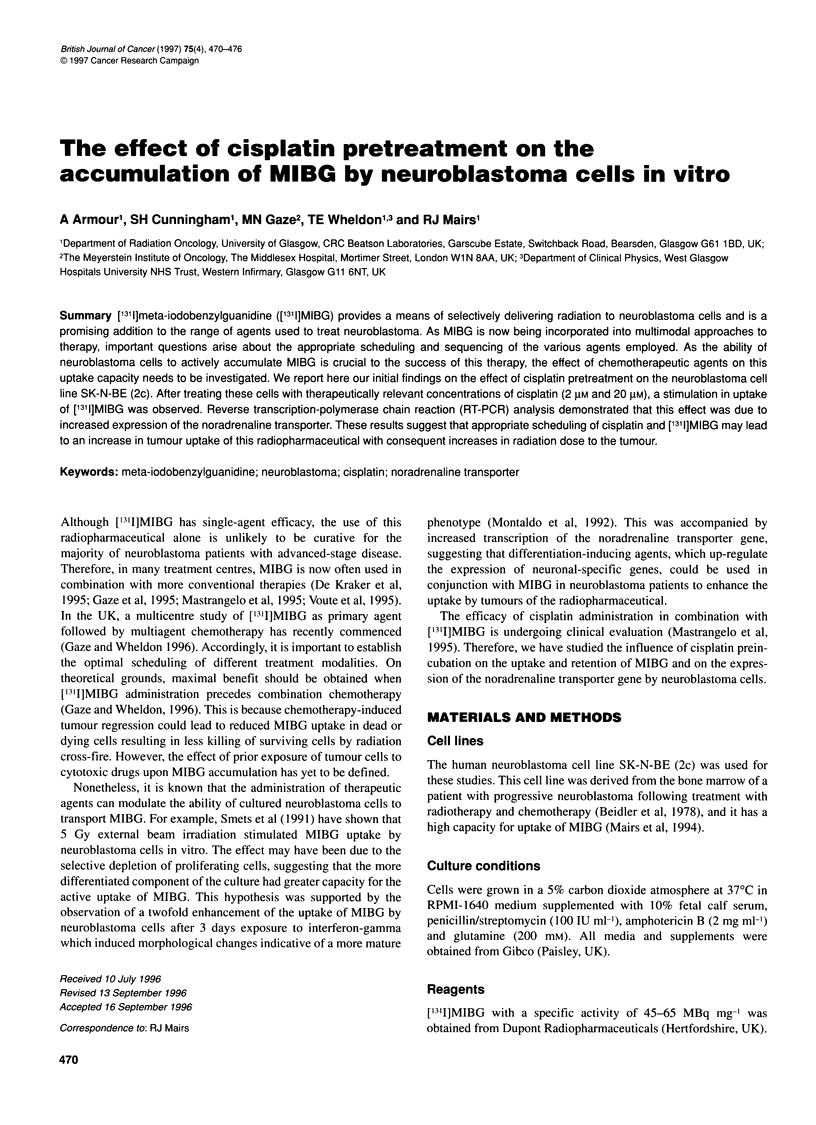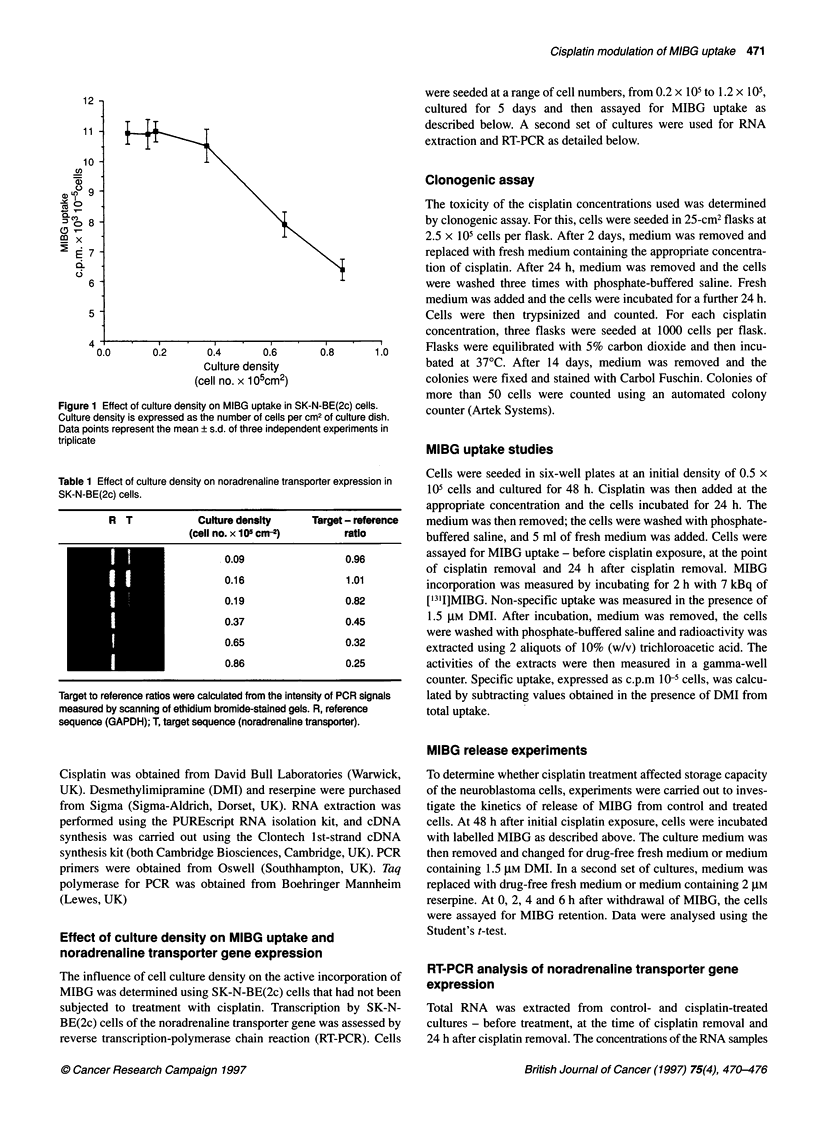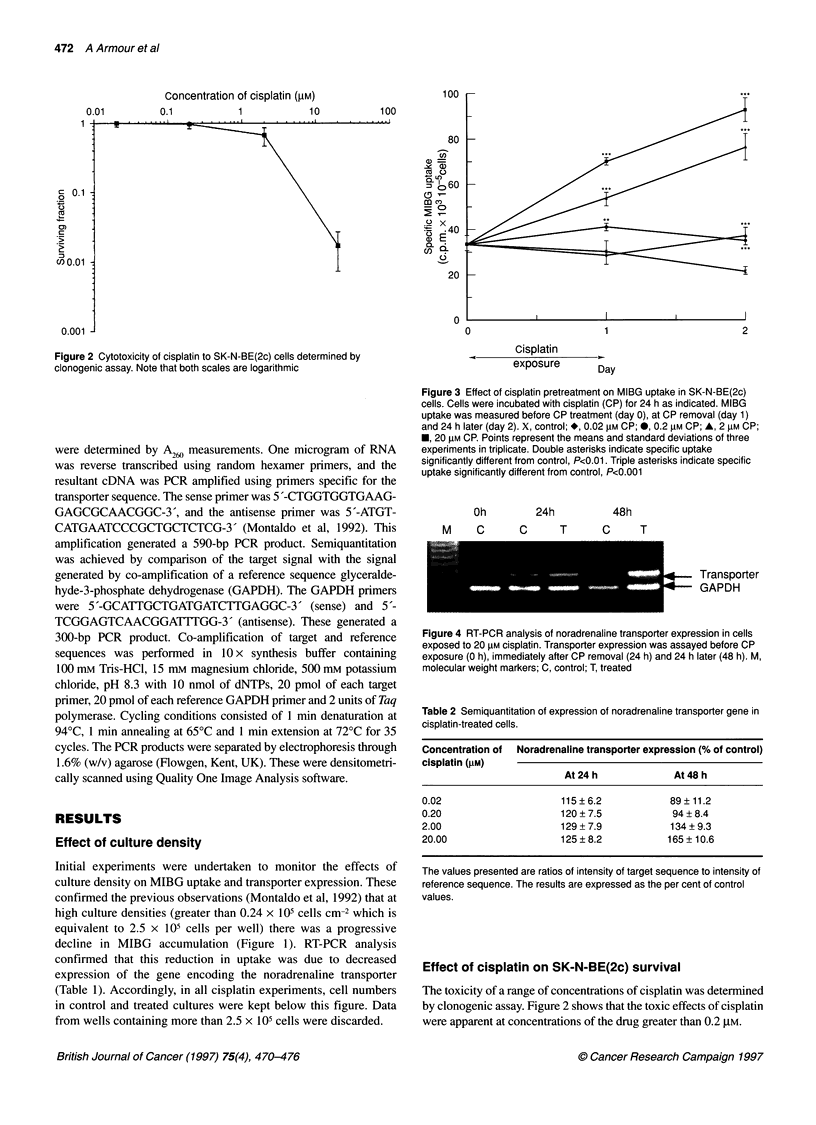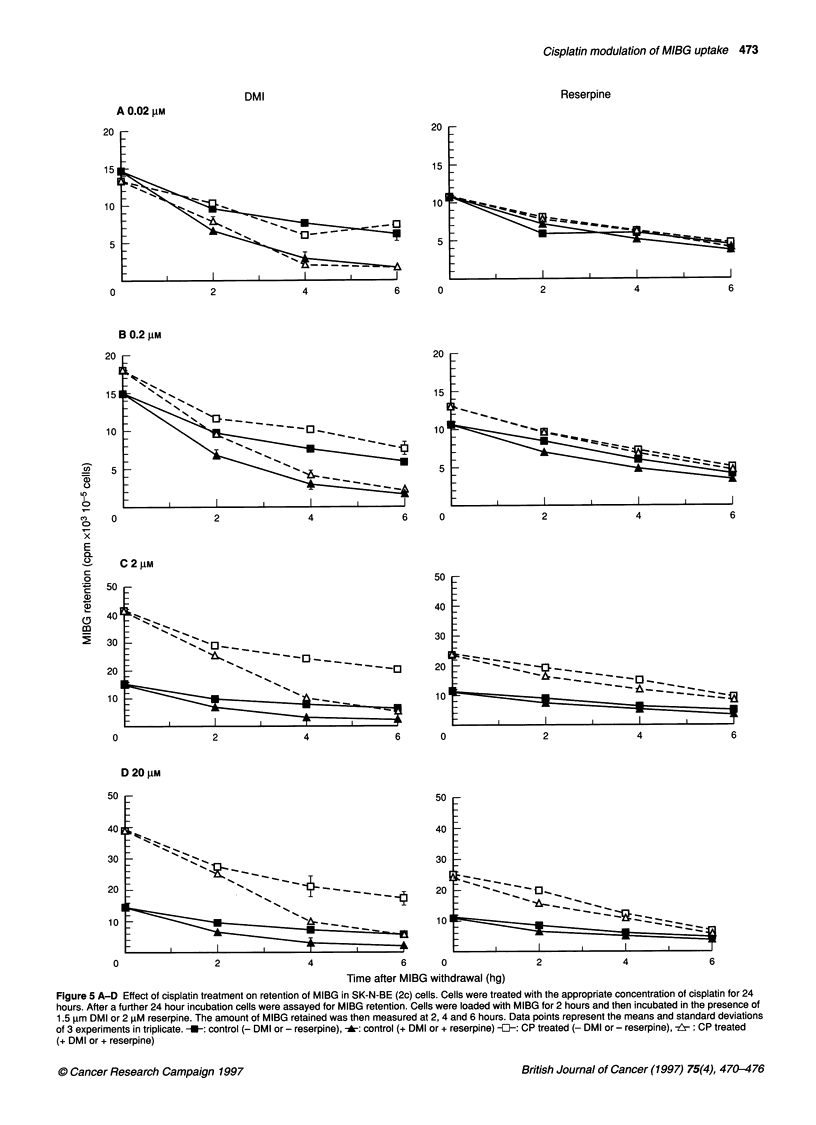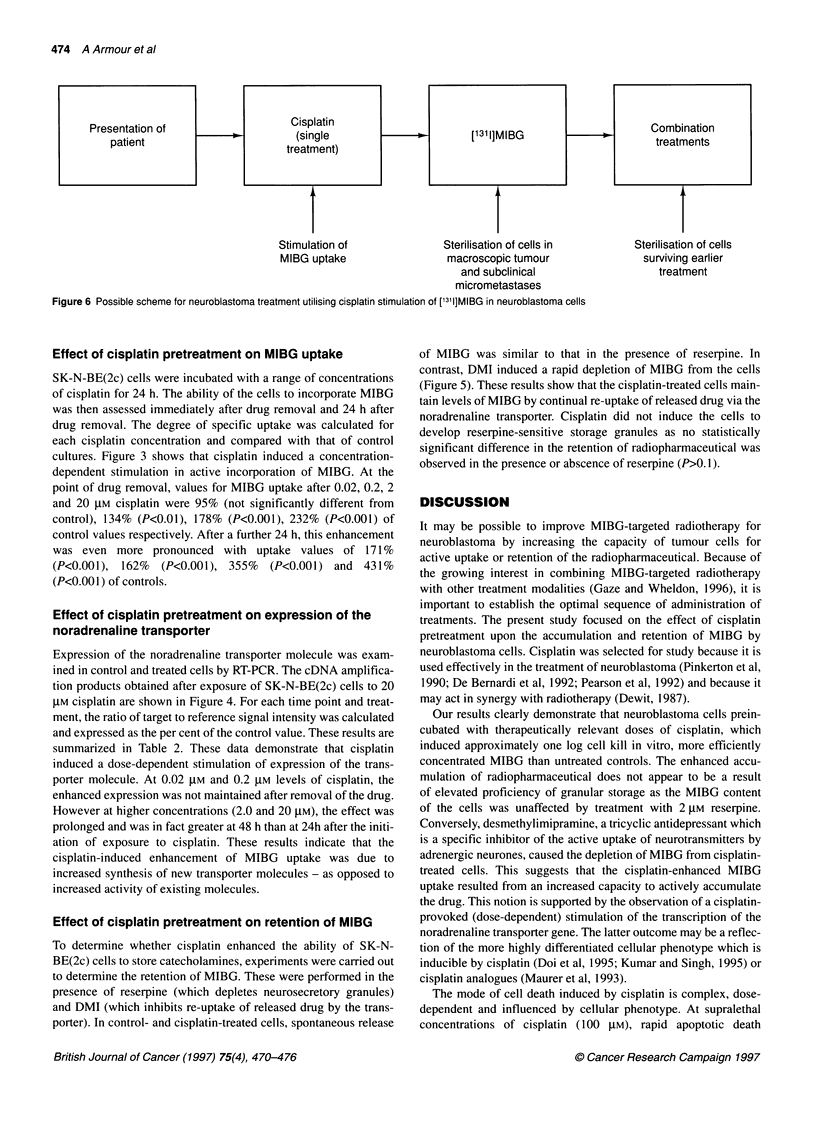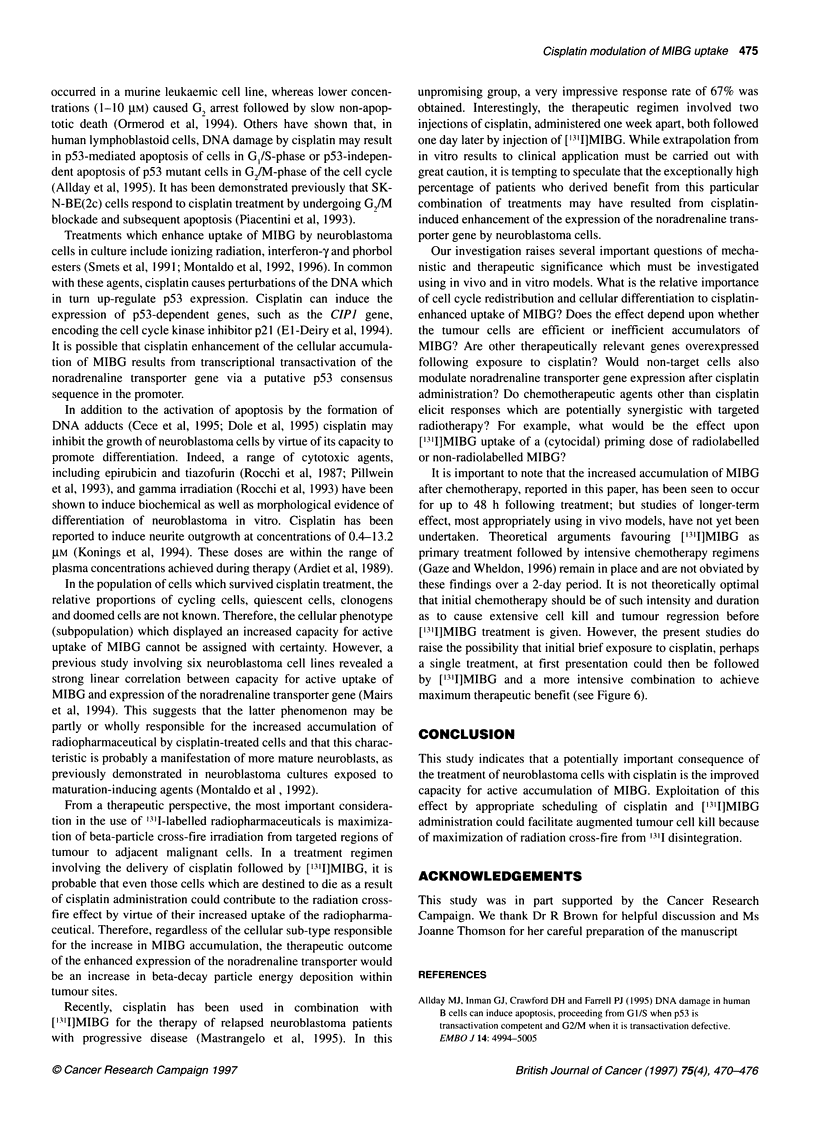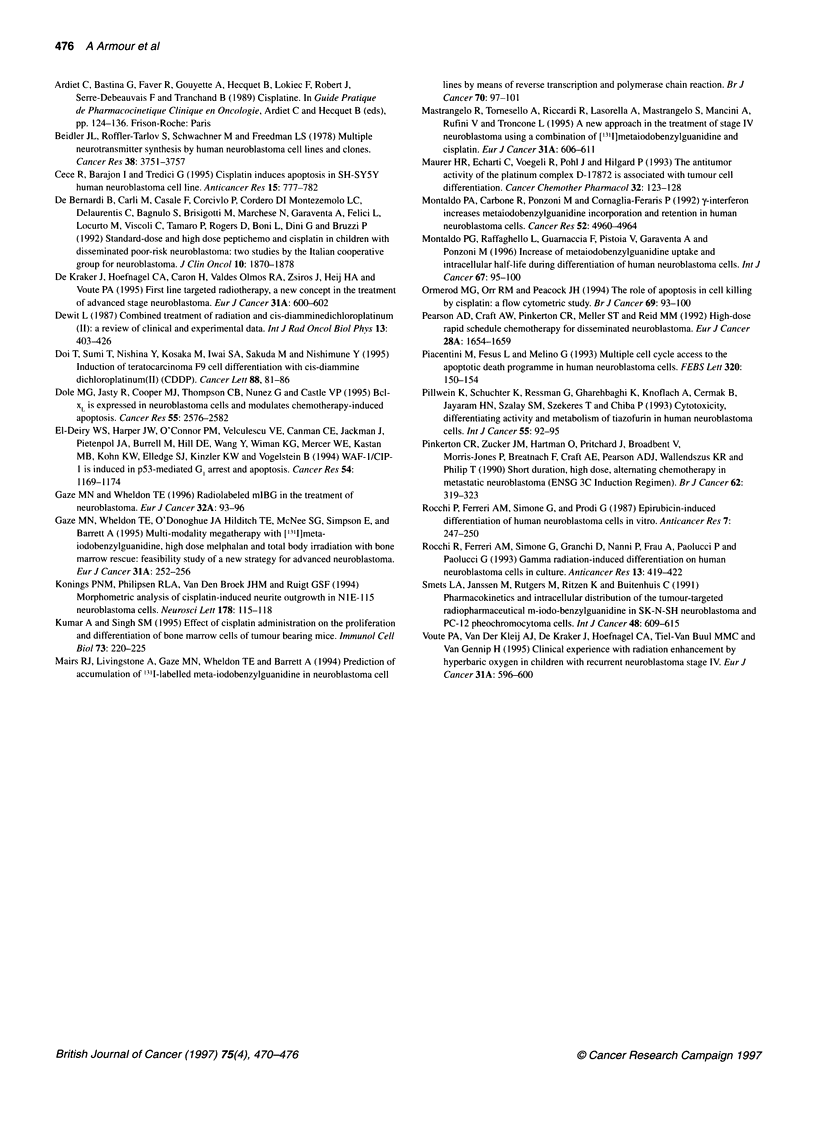# The effect of cisplatin pretreatment on the accumulation of MIBG by neuroblastoma cells in vitro.

**DOI:** 10.1038/bjc.1997.82

**Published:** 1997

**Authors:** A. Armour, S. H. Cunningham, M. N. Gaze, T. E. Wheldon, R. J. Mairs

**Affiliations:** Department of Radiation Oncology, University of Glasgow, CRC Beatson Laboratories, Bearsden, UK.

## Abstract

**Images:**


British Journal of Cancer (1997) 75(4), 470–476
© 1997 Cancer Research Campaign

### The effect of cisplatin pretreatment on the accumulation of MIBG by neuroblastoma cells in vitro

**A Armour[1], SH Cunningham[1], MN Gaze[2], TE Wheldon[1,3] and RJ Mairs[1]**

[1]Department of Radiation Oncology, University of Glasgow, CRC Beatson Laboratories, Garscube Estate, Switchback Road, Bearsden, Glasgow G61 1BD, UK; [2]The Meyerstein Institute of Oncology, The Middlesex Hospital, Mortimer Street, London W1N 8AA, UK; [3]Department of Clinical Physics, West Glasgow Hospitals University NHS Trust, Western Infirmary, Glasgow G11 6NT, UK

**Summary** [$^{131}$I]meta-iodobenzylguanidine ([$^{131}$I]MIBG) provides a means of selectively delivering radiation to neuroblastoma cells and is a promising addition to the range of agents used to treat neuroblastoma. As MIBG is now being incorporated into multimodal approaches to therapy, important questions arise about the appropriate scheduling and sequencing of the various agents employed. As the ability of neuroblastoma cells to actively accumulate MIBG is crucial to the success of this therapy, the effect of chemotherapeutic agents on this uptake capacity needs to be investigated. We report here our initial findings on the effect of cisplatin pretreatment on the neuroblastoma cell line SK-N-BE (2c). After treating these cells with therapeutically relevant concentrations of cisplatin (2 μM and 20 μM), a stimulation in uptake of [$^{131}$I]MIBG was observed. Reverse transcription-polymerase chain reaction (RT-PCR) analysis demonstrated that this effect was due to increased expression of the noradrenaline transporter. These results suggest that appropriate scheduling of cisplatin and [$^{131}$I]MIBG may lead to an increase in tumour uptake of this radiopharmaceutical with consequent increases in radiation dose to the tumour.

**Keywords:** meta-iodobenzylguanidine; neuroblastoma; cisplatin; noradrenaline transporter

Although [$^{131}$I]MIBG has single-agent efficacy, the use of this radiopharmaceutical alone is unlikely to be curative for the majority of neuroblastoma patients with advanced-stage disease. Therefore, in many treatment centres, MIBG is now often used in combination with more conventional therapies (De Kraker et al, 1995; Gaze et al, 1995; Mastrangelo et al, 1995; Voute et al, 1995). In the UK, a multicentre study of [$^{131}$I]MIBG as primary agent followed by multiagent chemotherapy has recently commenced (Gaze and Wheldon 1996). Accordingly, it is important to establish the optimal scheduling of different treatment modalities. On theoretical grounds, maximal benefit should be obtained when [$^{131}$I]MIBG administration precedes combination chemotherapy (Gaze and Wheldon, 1996). This is because chemotherapy-induced tumour regression could lead to reduced MIBG uptake in dead or dying cells resulting in less killing of surviving cells by radiation cross-fire. However, the effect of prior exposure of tumour cells to cytotoxic drugs upon MIBG accumulation has yet to be defined.

Nonetheless, it is known that the administration of therapeutic agents can modulate the ability of cultured neuroblastoma cells to transport MIBG. For example, Smets et al (1991) have shown that 5 Gy external beam irradiation stimulated MIBG uptake by neuroblastoma cells in vitro. The effect may have been due to the selective depletion of proliferating cells, suggesting that the more differentiated component of the culture had greater capacity for the active uptake of MIBG. This hypothesis was supported by the observation of a twofold enhancement of the uptake of MIBG by neuroblastoma cells after 3 days exposure to interferon-gamma which induced morphological changes indicative of a more mature phenotype (Montaldo et al, 1992). This was accompanied by increased transcription of the noradrenaline transporter gene, suggesting that differentiation-inducing agents, which up-regulate the expression of neuronal-specific genes, could be used in conjunction with MIBG in neuroblastoma patients to enhance the uptake by tumours of the radiopharmaceutical.

The efficacy of cisplatin administration in combination with [$^{131}$I]MIBG is undergoing clinical evaluation (Mastrangelo et al, 1995). Therefore, we have studied the influence of cisplatin preincubation on the uptake and retention of MIBG and on the expression of the noradrenaline transporter gene by neuroblastoma cells.

*Received 10 July 1996*
*Revised 13 September 1996*
*Accepted 16 September 1996*

*Correspondence to:* RJ Mairs

### MATERIALS AND METHODS

### Cell lines

The human neuroblastoma cell line SK-N-BE (2c) was used for these studies. This cell line was derived from the bone marrow of a patient with progressive neuroblastoma following treatment with radiotherapy and chemotherapy (Beidler et al, 1978), and it has a high capacity for uptake of MIBG (Mairs et al, 1994).

### Culture conditions

Cells were grown in a 5% carbon dioxide atmosphere at 37°C in RPMI-1640 medium supplemented with 10% fetal calf serum, penicillin/streptomycin (100 IU ml$^{-1}$), amphotericin B (2 mg ml$^{-1}$) and glutamine (200 mM). All media and supplements were obtained from Gibco (Paisley, UK).

### Reagents

[$^{131}$I]MIBG with a specific activity of 45–65 MBq mg$^{-1}$ was obtained from Dupont Radiopharmaceuticals (Hertfordshire, UK).

**Figure 1** Effect of culture density on MIBG uptake in SK-N-BE(2c) cells. Culture density is expressed as the number of cells per $cm^2$ of culture dish. Data points represent the mean ± s.d. of three independent experiments in triplicate

**Table 1** Effect of culture density on noradrenaline transporter expression in SK-N-BE(2c) cells.

| R T | Culture density (cell no. × $10^5$ $cm^{-2}$) | Target – reference ratio |
|---|---|---|
|  | 0.09 | 0.96 |
| | 0.16 | 1.01 |
| | 0.19 | 0.82 |
| | 0.37 | 0.45 |
| | 0.65 | 0.32 |
| | 0.86 | 0.25 |

Target to reference ratios were calculated from the intensity of PCR signals measured by scanning of ethidium bromide-stained gels. R, reference sequence (GAPDH); T, target sequence (noradrenaline transporter).

Cisplatin was obtained from David Bull Laboratories (Warwick, UK). Desmethylimipramine (DMI) and reserpine were purchased from Sigma (Sigma-Aldrich, Dorset, UK). RNA extraction was performed using the PUREscript RNA isolation kit, and cDNA synthesis was carried out using the Clontech 1st-strand cDNA synthesis kit (both Cambridge Biosciences, Cambridge, UK). PCR primers were obtained from Oswell (Southhampton, UK). *Taq* polymerase for PCR was obtained from Boehringer Mannheim (Lewes, UK)

### Effect of culture density on MIBG uptake and noradrenaline transporter gene expression

The influence of cell culture density on the active incorporation of MIBG was determined using SK-N-BE(2c) cells that had not been subjected to treatment with cisplatin. Transcription by SK-N-BE(2c) cells of the noradrenaline transporter gene was assessed by reverse transcription-polymerase chain reaction (RT-PCR). Cells were seeded at a range of cell numbers, from $0.2 \times 10^5$ to $1.2 \times 10^5$, cultured for 5 days and then assayed for MIBG uptake as described below. A second set of cultures were used for RNA extraction and RT-PCR as detailed below.

### Clonogenic assay

The toxicity of the cisplatin concentrations used was determined by clonogenic assay. For this, cells were seeded in 25-$cm^2$ flasks at $2.5 \times 10^5$ cells per flask. After 2 days, medium was removed and replaced with fresh medium containing the appropriate concentration of cisplatin. After 24 h, medium was removed and the cells were washed three times with phosphate-buffered saline. Fresh medium was added and the cells were incubated for a further 24 h. Cells were then trypsinized and counted. For each cisplatin concentration, three flasks were seeded at 1000 cells per flask. Flasks were equilibrated with 5% carbon dioxide and then incubated at 37°C. After 14 days, medium was removed and the colonies were fixed and stained with Carbol Fuschin. Colonies of more than 50 cells were counted using an automated colony counter (Artek Systems).

### MIBG uptake studies

Cells were seeded in six-well plates at an initial density of $0.5 \times 10^5$ cells and cultured for 48 h. Cisplatin was then added at the appropriate concentration and the cells incubated for 24 h. The medium was then removed; the cells were washed with phosphate-buffered saline, and 5 ml of fresh medium was added. Cells were assayed for MIBG uptake – before cisplatin exposure, at the point of cisplatin removal and 24 h after cisplatin removal. MIBG incorporation was measured by incubating for 2 h with 7 kBq of [$^{131}$I]MIBG. Non-specific uptake was measured in the presence of 1.5 μM DMI. After incubation, medium was removed, the cells were washed with phosphate-buffered saline and radioactivity was extracted using 2 aliquots of 10% (w/v) trichloroacetic acid. The activities of the extracts were then measured in a gamma-well counter. Specific uptake, expressed as c.p.m $10^{-5}$ cells, was calculated by subtracting values obtained in the presence of DMI from total uptake.

### MIBG release experiments

To determine whether cisplatin treatment affected storage capacity of the neuroblastoma cells, experiments were carried out to investigate the kinetics of release of MIBG from control and treated cells. At 48 h after initial cisplatin exposure, cells were incubated with labelled MIBG as described above. The culture medium was then removed and changed for drug-free fresh medium or medium containing 1.5 μM DMI. In a second set of cultures, medium was replaced with drug-free fresh medium or medium containing 2 μM reserpine. At 0, 2, 4 and 6 h after withdrawal of MIBG, the cells were assayed for MIBG retention. Data were analysed using the Student's *t*-test.

### RT-PCR analysis of noradrenaline transporter gene expression

Total RNA was extracted from control- and cisplatin-treated cultures – before treatment, at the time of cisplatin removal and 24 h after cisplatin removal. The concentrations of the RNA samples

© Cancer Research Campaign 1997 

**Figure 2** Cytotoxicity of cisplatin to SK-N-BE(2c) cells determined by clonogenic assay. Note that both scales are logarithmic

**Figure 3** Effect of cisplatin pretreatment on MIBG uptake in SK-N-BE(2c) cells. Cells were incubated with cisplatin (CP) for 24 h as indicated. MIBG uptake was measured before CP treatment (day 0), at CP removal (day 1) and 24 h later (day 2). X, control; ◆, 0.02 μM CP; ●, 0.2 μM CP; ▲, 2 μM CP; ■, 20 μM CP. Points represent the means and standard deviations of three experiments in triplicate. Double asterisks indicate specific uptake significantly different from control, $P<0.01$. Triple asterisks indicate specific uptake significantly different from control, $P<0.001$

were determined by $A_{260}$ measurements. One microgram of RNA was reverse transcribed using random hexamer primers, and the resultant cDNA was PCR amplified using primers specific for the transporter sequence. The sense primer was 5′-CTGGTGGTGAAG-GAGCGCAACGGC-3′, and the antisense primer was 5′-ATGT-CATGAATCCCGCTGCTCTCG-3′ (Montaldo et al, 1992). This amplification generated a 590-bp PCR product. Semiquantitation was achieved by comparison of the target signal with the signal generated by co-amplification of a reference sequence glyceraldehyde-3-phosphate dehydrogenase (GAPDH). The GAPDH primers were 5′-GCATTGCTGATGATCTTGAGGC-3′ (sense) and 5′-TCGGAGTCAACGGATTTGG-3′ (antisense). These generated a 300-bp PCR product. Co-amplification of target and reference sequences was performed in 10× synthesis buffer containing 100 mM Tris-HCl, 15 mM magnesium chloride, 500 mM potassium chloride, pH 8.3 with 10 nmol of dNTPs, 20 pmol of each target primer, 20 pmol of each reference GAPDH primer and 2 units of *Taq* polymerase. Cycling conditions consisted of 1 min denaturation at 94°C, 1 min annealing at 65°C and 1 min extension at 72°C for 35 cycles. The PCR products were separated by electrophoresis through 1.6% (w/v) agarose (Flowgen, Kent, UK). These were densitometrically scanned using Quality One Image Analysis software.

### RESULTS

### Effect of culture density

Initial experiments were undertaken to monitor the effects of culture density on MIBG uptake and transporter expression. These confirmed the previous observations (Montaldo et al, 1992) that at high culture densities (greater than $0.24 \times 10^5$ cells $cm^{-2}$ which is equivalent to $2.5 \times 10^5$ cells per well) there was a progressive decline in MIBG accumulation (Figure 1). RT-PCR analysis confirmed that this reduction in uptake was due to decreased expression of the gene encoding the noradrenaline transporter (Table 1). Accordingly, in all cisplatin experiments, cell numbers in control and treated cultures were kept below this figure. Data from wells containing more than $2.5 \times 10^5$ cells were discarded.

**Figure 4** RT-PCR analysis of noradrenaline transporter expression in cells exposed to 20 μM cisplatin. Transporter expression was assayed before CP exposure (0 h), immediately after CP removal (24 h) and 24 h later (48 h). M, molecular weight markers; C, control; T, treated

**Table 2** Semiquantitation of expression of noradrenaline transporter gene in cisplatin-treated cells.

| Concentration of cisplatin (μM) | Noradrenaline transporter expression (% of control) | |
|---|---|---|
| | At 24 h | At 48 h |
| 0.02 | 115 ± 6.2 | 89 ± 11.2 |
| 0.20 | 120 ± 7.5 | 94 ± 8.4 |
| 2.00 | 129 ± 7.9 | 134 ± 9.3 |
| 20.00 | 125 ± 8.2 | 165 ± 10.6 |

The values presented are ratios of intensity of target sequence to intensity of reference sequence. The results are expressed as the per cent of control values.

### Effect of cisplatin on SK-N-BE(2c) survival

The toxicity of a range of concentrations of cisplatin was determined by clonogenic assay. Figure 2 shows that the toxic effects of cisplatin were apparent at concentrations of the drug greater than 0.2 μM.

© Cancer Research Campaign 1997

**Figure 5 A–D** Effect of cisplatin treatment on retention of MIBG in SK-N-BE (2c) cells. Cells were treated with the appropriate concentration of cisplatin for 24 hours. After a further 24 hour incubation cells were assayed for MIBG retention. Cells were loaded with MIBG for 2 hours and then incubated in the presence of 1.5 μm DMI or 2 μM reserpine. The amount of MIBG retained was then measured at 2, 4 and 6 hours. Data points represent the means and standard deviations of 3 experiments in triplicate. -■-: control (– DMI or – reserpine), -▲-: control (+ DMI or + reserpine) -□-: CP treated (– DMI or – reserpine), -△- : CP treated (+ DMI or + reserpine)

**Figure 6** Possible scheme for neuroblastoma treatment utilising cisplatin stimulation of [$^{131}$I]MIBG in neuroblastoma cells

### Effect of cisplatin pretreatment on MIBG uptake

SK-N-BE(2c) cells were incubated with a range of concentrations of cisplatin for 24 h. The ability of the cells to incorporate MIBG was then assessed immediately after drug removal and 24 h after drug removal. The degree of specific uptake was calculated for each cisplatin concentration and compared with that of control cultures. Figure 3 shows that cisplatin induced a concentration-dependent stimulation in active incorporation of MIBG. At the point of drug removal, values for MIBG uptake after 0.02, 0.2, 2 and 20 μM cisplatin were 95% (not significantly different from control), 134% ($P$<0.01), 178% ($P$<0.001), 232% ($P$<0.001) of control values respectively. After a further 24 h, this enhancement was even more pronounced with uptake values of 171% ($P$<0.001), 162% ($P$<0.001), 355% ($P$<0.001) and 431% ($P$<0.001) of controls.

### Effect of cisplatin pretreatment on expression of the noradrenaline transporter

Expression of the noradrenaline transporter molecule was examined in control and treated cells by RT-PCR. The cDNA amplification products obtained after exposure of SK-N-BE(2c) cells to 20 μM cisplatin are shown in Figure 4. For each time point and treatment, the ratio of target to reference signal intensity was calculated and expressed as the per cent of the control value. These results are summarized in Table 2. These data demonstrate that cisplatin induced a dose-dependent stimulation of expression of the transporter molecule. At 0.02 μM and 0.2 μM levels of cisplatin, the enhanced expression was not maintained after removal of the drug. However at higher concentrations (2.0 and 20 μM), the effect was prolonged and was in fact greater at 48 h than at 24h after the initiation of exposure to cisplatin. These results indicate that the cisplatin-induced enhancement of MIBG uptake was due to increased synthesis of new transporter molecules – as opposed to increased activity of existing molecules.

### Effect of cisplatin pretreatment on retention of MIBG

To determine whether cisplatin enhanced the ability of SK-N-BE(2c) cells to store catecholamines, experiments were carried out to determine the retention of MIBG. These were performed in the presence of reserpine (which depletes neurosecretory granules) and DMI (which inhibits re-uptake of released drug by the transporter). In control- and cisplatin-treated cells, spontaneous release of MIBG was similar to that in the presence of reserpine. In contrast, DMI induced a rapid depletion of MIBG from the cells (Figure 5). These results show that the cisplatin-treated cells maintain levels of MIBG by continual re-uptake of released drug via the noradrenaline transporter. Cisplatin did not induce the cells to develop reserpine-sensitive storage granules as no statistically significant difference in the retention of radiopharmaceutical was observed in the presence or abscence of reserpine ($P$>0.1).

### DISCUSSION

It may be possible to improve MIBG-targeted radiotherapy for neuroblastoma by increasing the capacity of tumour cells for active uptake or retention of the radiopharmaceutical. Because of the growing interest in combining MIBG-targeted radiotherapy with other treatment modalities (Gaze and Wheldon, 1996), it is important to establish the optimal sequence of administration of treatments. The present study focused on the effect of cisplatin pretreatment upon the accumulation and retention of MIBG by neuroblastoma cells. Cisplatin was selected for study because it is used effectively in the treatment of neuroblastoma (Pinkerton et al, 1990; De Bernardi et al, 1992; Pearson et al, 1992) and because it may act in synergy with radiotherapy (Dewit, 1987).

Our results clearly demonstrate that neuroblastoma cells preincubated with therapeutically relevant doses of cisplatin, which induced approximately one log cell kill in vitro, more efficiently concentrated MIBG than untreated controls. The enhanced accumulation of radiopharmaceutical does not appear to be a result of elevated proficiency of granular storage as the MIBG content of the cells was unaffected by treatment with 2 μM reserpine. Conversely, desmethylimipramine, a tricyclic antidepressant which is a specific inhibitor of the active uptake of neurotransmitters by adrenergic neurones, caused the depletion of MIBG from cisplatin-treated cells. This suggests that the cisplatin-enhanced MIBG uptake resulted from an increased capacity to actively accumulate the drug. This notion is supported by the observation of a cisplatin-provoked (dose-dependent) stimulation of the transcription of the noradrenaline transporter gene. The latter outcome may be a reflection of the more highly differentiated cellular phenotype which is inducible by cisplatin (Doi et al, 1995; Kumar and Singh, 1995) or cisplatin analogues (Maurer et al, 1993).

The mode of cell death induced by cisplatin is complex, dose-dependent and influenced by cellular phenotype. At supralethal concentrations of cisplatin (100 μM), rapid apoptotic death

© Cancer Research Campaign 1997

occurred in a murine leukaemic cell line, whereas lower concentrations (1–10 μM) caused $G_2$ arrest followed by slow non-apoptotic death (Ormerod et al, 1994). Others have shown that, in human lymphoblastoid cells, DNA damage by cisplatin may result in p53-mediated apoptosis of cells in $G_1$/S-phase or p53-independent apoptosis of p53 mutant cells in $G_2$/M-phase of the cell cycle (Allday et al, 1995). It has been demonstrated previously that SK-N-BE(2c) cells respond to cisplatin treatment by undergoing $G_2$/M blockade and subsequent apoptosis (Piacentini et al, 1993).

Treatments which enhance uptake of MIBG by neuroblastoma cells in culture include ionizing radiation, interferon-γ and phorbol esters (Smets et al, 1991; Montaldo et al, 1992, 1996). In common with these agents, cisplatin causes perturbations of the DNA which in turn up-regulate p53 expression. Cisplatin can induce the expression of p53-dependent genes, such as the *CIP1* gene, encoding the cell cycle kinase inhibitor p21 (El-Deiry et al, 1994). It is possible that cisplatin enhancement of the cellular accumulation of MIBG results from transcriptional transactivation of the noradrenaline transporter gene via a putative p53 consensus sequence in the promoter.

In addition to the activation of apoptosis by the formation of DNA adducts (Cece et al, 1995; Dole et al, 1995) cisplatin may inhibit the growth of neuroblastoma cells by virtue of its capacity to promote differentiation. Indeed, a range of cytotoxic agents, including epirubicin and tiazofurin (Rocchi et al, 1987; Pillwein et al, 1993), and gamma irradiation (Rocchi et al, 1993) have been shown to induce biochemical as well as morphological evidence of differentiation of neuroblastoma in vitro. Cisplatin has been reported to induce neurite outgrowth at concentrations of 0.4–13.2 μM (Konings et al, 1994). These doses are within the range of plasma concentrations achieved during therapy (Ardiet et al, 1989).

In the population of cells which survived cisplatin treatment, the relative proportions of cycling cells, quiescent cells, clonogens and doomed cells are not known. Therefore, the cellular phenotype (subpopulation) which displayed an increased capacity for active uptake of MIBG cannot be assigned with certainty. However, a previous study involving six neuroblastoma cell lines revealed a strong linear correlation between capacity for active uptake of MIBG and expression of the noradrenaline transporter gene (Mairs et al, 1994). This suggests that the latter phenomenon may be partly or wholly responsible for the increased accumulation of radiopharmaceutical by cisplatin-treated cells and that this characteristic is probably a manifestation of more mature neuroblasts, as previously demonstrated in neuroblastoma cultures exposed to maturation-inducing agents (Montaldo et al , 1992).

From a therapeutic perspective, the most important consideration in the use of $^{131}$I-labelled radiopharmaceuticals is maximization of beta-particle cross-fire irradiation from targeted regions of tumour to adjacent malignant cells. In a treatment regimen involving the delivery of cisplatin followed by [$^{131}$I]MIBG, it is probable that even those cells which are destined to die as a result of cisplatin administration could contribute to the radiation cross-fire effect by virtue of their increased uptake of the radiopharmaceutical. Therefore, regardless of the cellular sub-type responsible for the increase in MIBG accumulation, the therapeutic outcome of the enhanced expression of the noradrenaline transporter would be an increase in beta-decay particle energy deposition within tumour sites.

Recently, cisplatin has been used in combination with [$^{131}$I]MIBG for the therapy of relapsed neuroblastoma patients with progressive disease (Mastrangelo et al, 1995). In this unpromising group, a very impressive response rate of 67% was obtained. Interestingly, the therapeutic regimen involved two injections of cisplatin, administered one week apart, both followed one day later by injection of [$^{131}$I]MIBG. While extrapolation from in vitro results to clinical application must be carried out with great caution, it is tempting to speculate that the exceptionally high percentage of patients who derived benefit from this particular combination of treatments may have resulted from cisplatin-induced enhancement of the expression of the noradrenaline transporter gene by neuroblastoma cells.

Our investigation raises several important questions of mechanistic and therapeutic significance which must be investigated using in vivo and in vitro models. What is the relative importance of cell cycle redistribution and cellular differentiation to cisplatin-enhanced uptake of MIBG? Does the effect depend upon whether the tumour cells are efficient or inefficient accumulators of MIBG? Are other therapeutically relevant genes overexpressed following exposure to cisplatin? Would non-target cells also modulate noradrenaline transporter gene expression after cisplatin administration? Do chemotherapeutic agents other than cisplatin elicit responses which are potentially synergistic with targeted radiotherapy? For example, what would be the effect upon [$^{131}$I]MIBG uptake of a (cytocidal) priming dose of radiolabelled or non-radiolabelled MIBG?

It is important to note that the increased accumulation of MIBG after chemotherapy, reported in this paper, has been seen to occur for up to 48 h following treatment; but studies of longer-term effect, most appropriately using in vivo models, have not yet been undertaken. Theoretical arguments favouring [$^{131}$I]MIBG as primary treatment followed by intensive chemotherapy regimens (Gaze and Wheldon, 1996) remain in place and are not obviated by these findings over a 2-day period. It is not theoretically optimal that initial chemotherapy should be of such intensity and duration as to cause extensive cell kill and tumour regression before [$^{131}$I]MIBG treatment is given. However, the present studies do raise the possibility that initial brief exposure to cisplatin, perhaps a single treatment, at first presentation could then be followed by [$^{131}$I]MIBG and a more intensive combination to achieve maximum therapeutic benefit (see Figure 6).

### CONCLUSION

This study indicates that a potentially important consequence of the treatment of neuroblastoma cells with cisplatin is the improved capacity for active accumulation of MIBG. Exploitation of this effect by appropriate scheduling of cisplatin and [$^{131}$I]MIBG administration could facilitate augmented tumour cell kill because of maximization of radiation cross-fire from $^{131}$I disintegration.

### ACKNOWLEDGEMENTS

This study was in part supported by the Cancer Research Campaign. We thank Dr R Brown for helpful discussion and Ms Joanne Thomson for her careful preparation of the manuscript

### REFERENCES

Allday MJ, Inman GJ, Crawford DH and Farrell PJ (1995) DNA damage in human B cells can induce apoptosis, proceeding from G1/S when p53 is transactivation competent and G2/M when it is transactivation defective. *EMBO J* **14**: 4994–5005

© Cancer Research Campaign 1997 

Ardiet C, Bastina G, Faver R, Gouyette A, Hecquet B, Lokiec F, Robert J, Serre-Debeauvais F and Tranchand B (1989) Cisplatine. In *Guide Pratique de Pharmacocinetique Clinique en Oncologie*, Ardiet C and Hecquet B (eds), pp. 124–136. Frison-Roche: Paris

Beidler JL, Roffler-Tarlov S, Schwachner M and Freedman LS (1978) Multiple neurotransmitter synthesis by human neuroblastoma cell lines and clones. *Cancer Res* **38**: 3751–3757

Cece R, Barajon I and Tredici G (1995) Cisplatin induces apoptosis in SH-SY5Y human neuroblastoma cell line. *Anticancer Res* **15**: 777–782

De Bernardi B, Carli M, Casale F, Corcivlo P, Cordero DI Montezemolo LC, Delaurentis C, Bagnulo S, Brisigotti M, Marchese N, Garaventa A, Felici L, Locurto M, Viscoli C, Tamaro P, Rogers D, Boni L, Dini G and Bruzzi P (1992) Standard-dose and high dose peptichemo and cisplatin in children with disseminated poor-risk neuroblastoma: two studies by the Italian cooperative group for neuroblastoma. *J Clin Oncol* **10**: 1870–1878

De Kraker J, Hoefnagel CA, Caron H, Valdes Olmos RA, Zsiros J, Heij HA and Voute PA (1995) First line targeted radiotherapy, a new concept in the treatment of advanced stage neuroblastoma. *Eur J Cancer* **31A**: 600–602

Dewit L (1987) Combined treatment of radiation and cis-diamminedichloroplatinum (II): a review of clinical and experimental data. *Int J Rad Oncol Biol Phys* **13**: 403–426

Doi T, Sumi T, Nishina Y, Kosaka M, Iwai SA, Sakuda M and Nishimune Y (1995) Induction of teratocarcinoma F9 cell differentiation with cis-diammine dichloroplatinum(II) (CDDP). *Cancer Lett* **88**, 81–86

Dole MG, Jasty R, Cooper MJ, Thompson CB, Nunez G and Castle VP (1995) Bcl-$x_L$ is expressed in neuroblastoma cells and modulates chemotherapy-induced apoptosis. *Cancer Res* **55**: 2576–2582

El-Deiry WS, Harper JW, O'Connor PM, Velculescu VE, Canman CE, Jackman J, Pietenpol JA, Burrell M, Hill DE, Wang Y, Wiman KG, Mercer WE, Kastan MB, Kohn KW, Elledge SJ, Kinzler KW and Vogelstein B (1994) WAF-1/CIP-1 is induced in p53-mediated $G_1$ arrest and apoptosis. *Cancer Res* **54**: 1169–1174

Gaze MN and Wheldon TE (1996) Radiolabeled mIBG in the treatment of neuroblastoma. *Eur J Cancer* **32A**: 93–96

Gaze MN, Wheldon TE, O'Donoghue JA Hilditch TE, McNee SG, Simpson E, and Barrett A (1995) Multi-modality megatherapy with [$^{131}$I]meta-iodobenzylguanidine, high dose melphalan and total body irradiation with bone marrow rescue: feasibility study of a new strategy for advanced neuroblastoma. *Eur J Cancer* **31A**: 252–256

Konings PNM, Philipsen RLA, Van Den Broek JHM and Ruigt GSF (1994) Morphometric analysis of cisplatin-induced neurite outgrowth in N1E-115 neuroblastoma cells. *Neurosci Lett* **178**: 115–118

Kumar A and Singh SM (1995) Effect of cisplatin administration on the proliferation and differentiation of bone marrow cells of tumour bearing mice. *Immunol Cell Biol* **73**: 220–225

Mairs RJ, Livingstone A, Gaze MN, Wheldon TE and Barrett A (1994) Prediction of accumulation of $^{131}$I-labelled meta-iodobenzylguanidine in neuroblastoma cell lines by means of reverse transcription and polymerase chain reaction. *Br J Cancer* **70**: 97–101

Mastrangelo R, Tornesello A, Riccardi R, Lasorella A, Mastrangelo S, Mancini A, Rufini V and Troncone L (1995) A new approach in the treatment of stage IV neuroblastoma using a combination of [$^{131}$I]metaiodobenzylguanidine and cisplatin. *Eur J Cancer* **31A**: 606–611

Maurer HR, Echarti C, Voegeli R, Pohl J and Hilgard P (1993) The antitumor activity of the platinum complex D-17872 is associated with tumour cell differentiation. *Cancer Chemother Pharmacol* **32**: 123–128

Montaldo PA, Carbone R, Ponzoni M and Cornaglia-Feraris P (1992) γ-interferon increases metaiodobenzylguanidine incorporation and retention in human neuroblastoma cells. *Cancer Res* **52**: 4960–4964

Montaldo PG, Raffaghello L, Guarnaccia F, Pistoia V, Garaventa A and Ponzoni M (1996) Increase of metaiodobenzylguanidine uptake and intracellular half-life during differentiation of human neuroblastoma cells. *Int J Cancer* **67**: 95–100

Ormerod MG, Orr RM and Peacock JH (1994) The role of apoptosis in cell killing by cisplatin: a flow cytometric study. *Br J Cancer* **69**: 93–100

Pearson AD, Craft AW, Pinkerton CR, Meller ST and Reid MM (1992) High-dose rapid schedule chemotherapy for disseminated neuroblastoma. *Eur J Cancer* **28A**: 1654–1659

Piacentini M, Fesus L and Melino G (1993) Multiple cell cycle access to the apoptotic death programme in human neuroblastoma cells. *FEBS Lett* **320**: 150–154

Pillwein K, Schuchter K, Ressman G, Gharehbaghi K, Knoflach A, Cermak B, Jayaram HN, Szalay SM, Szekeres T and Chiba P (1993) Cytotoxicity, differentiating activity and metabolism of tiazofurin in human neuroblastoma cells. *Int J Cancer* **55**: 92–95

Pinkerton CR, Zucker JM, Hartman O, Pritchard J, Broadbent V, Morris-Jones P, Breatnach F, Craft AE, Pearson ADJ, Wallendszus KR and Philip T (1990) Short duration, high dose, alternating chemotherapy in metastatic neuroblastoma (ENSG 3C Induction Regimen). *Br J Cancer* **62**: 319–323

Rocchi P, Ferreri AM, Simone G, and Prodi G (1987) Epirubicin-induced differentiation of human neuroblastoma cells in vitro. *Anticancer Res* **7**: 247–250

Rocchi R, Ferreri AM, Simone G, Granchi D, Nanni P, Frau A, Paolucci P and Paolucci G (1993) Gamma radiation-induced differentiation on human neuroblastoma cells in culture. *Anticancer Res* **13**: 419–422

Smets LA, Janssen M, Rutgers M, Ritzen K and Buitenhuis C (1991) Pharmacokinetics and intracellular distribution of the tumour-targeted radiopharmaceutical m-iodo-benzylguanidine in SK-N-SH neuroblastoma and PC-12 pheochromocytoma cells. *Int J Cancer* **48**: 609–615

Voute PA, Van Der Kleij AJ, De Kraker J, Hoefnagel CA, Tiel-Van Buul MMC and Van Gennip H (1995) Clinical experience with radiation enhancement by hyperbaric oxygen in children with recurrent neuroblastoma stage IV. *Eur J Cancer* **31A**: 596–600

© Cancer Research Campaign 1997